# Design and 3D-printing of MRI-compatible cradle for imaging mouse tumors

**DOI:** 10.1186/s41205-021-00124-6

**Published:** 2021-10-19

**Authors:** Deborah L. Donohoe, Katherine Dennert, Rajeev Kumar, Bonnie P. Freudinger, Alexander J. Sherman

**Affiliations:** 1 Advocate Aurora Research Institute, Advocate Aurora Health Care, 960 N. 12th Street, Milwaukee, WI 53233 USA; 2 MCW Engineering Core, Medical College of Wisconsin, 8701 Watertown Plank Rd, Milwaukee, WI 53226 USA

**Keywords:** 3D printing, Low field MRI, MRI cradle, Stereotaxic device, Mouse imaging

## Abstract

**Background:**

The ability of 3D printing using plastics and resins that are magnetic resonance imaging (MRI) compatible provides opportunities to tailor design features to specific imaging needs. In this study an MRI compatible cradle was designed to fit the need for repeatable serial images of mice within a mouse specific low field MRI.

**Methods:**

Several designs were reviewed which resulted in an open style stereotaxic cradle to fit within specific bore tolerances and allow maximum flexibility with interchangeable radiofrequency (RF) coils. CAD drawings were generated, cradle was printed and tested with phantom material and animals. Images were analyzed for quality and optimized using the new cradle.

Testing with multiple phantoms was done to affirm that material choice did not create unwanted image artifact and to optimize imaging parameters. Once phantom testing was satisfied, mouse imaging began.

**Results:**

The 3D printed cradle fit instrument tolerances, accommodated multiple coil configurations and physiological monitoring equipment, and allowed for improved image quality and reproducibility while also reducing overall imaging time and animal safety.

**Conclusions:**

The generation of a 3D printed stereotaxic cradle was a low-cost option which functioned well for our laboratory.

## Introduction

Since its introduction in the 1980’s, three-dimensional (3D) printing, also known as rapid prototyping, has grown to provide solutions to a myriad of design challenges. With addition of CAD software, 3D printing has undergone a metamorphosis and is used in a wide range of medical applications including patient-specific anatomical modeling, surgical planning, medical implants, and patient education [1], and could decrease economic impact [2, 3]. Along with computed tomography (CT) and MRI, CAD software can be used in 3D printing to generate geometry for production models and devices [4, 5]. A cradle with specific geometries and features not commercially available was successfully designed, 3D printed, and implemented in this study. A valuable tool for small laboratories and limited budgets, rapid prototyping lends itself to production of specialty items that cannot be created using traditional fabrication techniques.

## Background

Using MRI for animal research provides a wealth of information regarding an animal’s condition without the need for dissection [6], decreasing the number of animals needed for an experimental protocol by allowing repeated measures on the same animal. MRI is also a relatively safe technique compared to CT as MRI does not require radiation to produce images [7].

3D printing is increasingly used to produce custom components for MRI. Often pre-manufactured components fail to meet specific geometric requirements for use in specialized imaging equipment, which can result in poor fit [8] and lead to low image quality. Creating a custom apparatus to fit the animal species allows for consistent position and reproducible images. This, in turn, creates consistent image quality for direct comparison [9]. In addition, scans can be performed more quickly as less time is needed to position the animal, which reduces anesthesia time and provides a safer environment for the animal [10].

### Animal safety

Rodents have a high metabolic rate compared to humans and require monitoring during procedures when anesthesia is used [11]. To ensure animal health during imaging, several variables are monitored including oxygen saturation, heart rate and body temperature. Inhalation anesthesia during MRI for mice is relatively safe; however, changes in respiration and oxygenation during the imaging protocol may affect image quality. When designing new equipment, consideration must be given to the anesthesia delivery mechanism as well as physiologic monitoring equipment used during imaging.

### In-house MRI unit

**Fig. 1:**
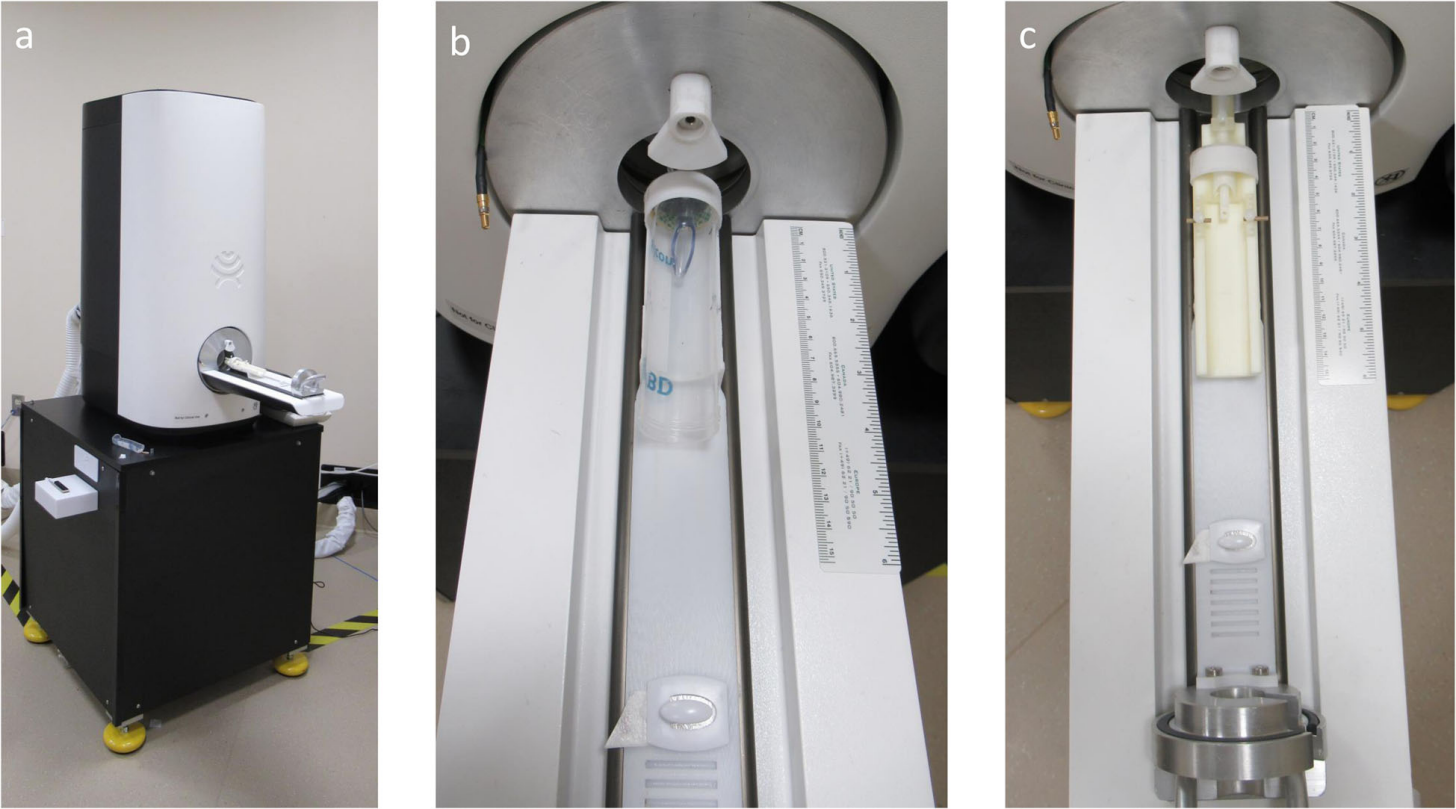
Equipment used in mouse magnetic resonance imaging. **a**. Synaptive 0.5 T Scout MRI. **b**. Homemade cradle system used for securing mice during imaging; centrifuge tube with drilled hole in top to accommodate inhalation anesthesia tubing and nose cone. **c**. 3D printed cradle

In a research setting, equipment and tools may be project- or program-specific. For example, this neuro-oncology research team uses a low field MRI (0.5 T Scout, Synaptive Medical, Toronto, Canada) (Fig. 1). This one-of-a-kind beta-test unit was designed specifically to study glioblastoma multiforme in a mouse (up to 30 g) model. The MRI is equipped with a small diameter imaging bore (3 cm), making commercially available animal positioning equipment inadequate to provide consistent, reproducible imaging with simultaneous delivery of inhaled anesthetic and physiologic monitoring while also accommodating multiple coil sizes. Unlike most MR imagers, the 0.5 T Scout has interchangeable receiver coils, rather than fixed coils within the sled apparatus, allowing for image acquisition flexibility.

### Design needs and challenges

Given our need for an MRI compatible cradle for the custom 3-cm bore, a search was undertaken to assess options and designs that met requirements for imaging brain and other tumors using this unique imager. The primary cradle device requirements included: non-ferric MRI compatible material, fit within the 3-cm bore, ability to accommodate various coil sizes, and ability to achieve and maintain consistent mouse position. No commercially available devices met the size requirements for the 3-cm bore. These challenges presented an opportunity to design and create a custom 3D printed apparatus to fit our needs.

Many MRI devices designed to place the animal in a fixed position, are designed in a cylindrical form [12] and incorporate a moveable sled, cradle and receiver coil. This design limits function and requires additional animal manipulation or a different cradle to obtain images using a different coil. The small-bore size of the 0.5 T Scout MRI and interchangeable coil configurations dictated the overall design requirements.

## Materials and methods

### 3D printing prototype investigation, development, and testing

**Fig. 2:**
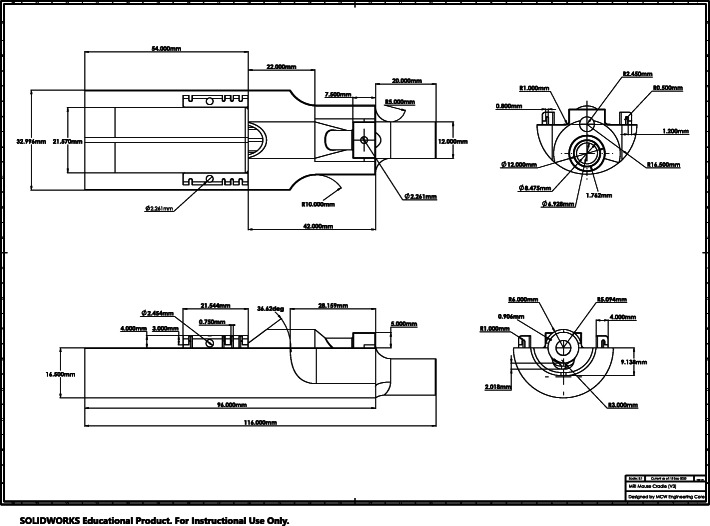
3D printed cradle schematic. Top view illustrates cradle bed and nose cone. Side view illustrates opening for bite bar and anesthesia delivery tunnel

3D printing of an open cradle design was determined to be the best approach to fit the space requirements and accommodate consistent mouse position, various coil configurations and physiologic monitoring equipment. The open cradle design incorporates stereotaxic positioning with a bite bar, ear bars and anesthesia nosecone. A tapered end was desired to fit into the existing sled, provide flexibility for interchangeable RF coils and allow consistent animal position while under anesthesia. CAD drawings (Fig. 2) were generated in Solidworks design software based on the device requirements and desired features. After it was determined that all design criteria were met and the cost to generate the MRI cradle was established, the device was approved for printing.

**Fig. 3:**
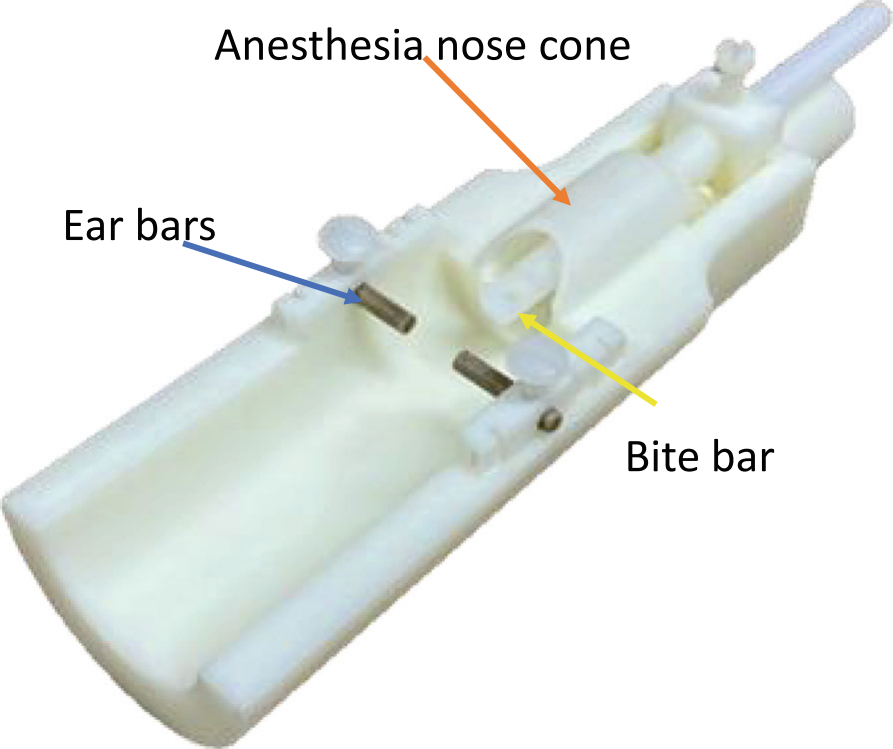
3D printed cradle. Stereotaxic design includes ear bars (blue arrow), bite bar (yellow arrow), and anesthesia delivery nose cone (orange arrow) to fit inside the 3 cm bore

The protype device was printed using MultiJet Printing technology (ProJet 3500 HDMax, 3D Systems, Rock Hill, SC) in Xtreme High Definition (XHD) mode (Fig. 3).

A proprietary plastic material (VisiJet M3-X, 3D Systems) was printed at a 16-μm print resolution with S-300 support material (Table 1).

**Table 1:** Physical properties of the M3-X proprietary plastic resin as provided by 3D systems

Properties	Condition	VisiJet M3-X
Color		White
Density @ 80 degrees Celsius		1.04 g/cm^3^
Tensile Strength	ASTM D638	49 MPa
Tensile Modulus	ASTM D638	2168 MPa
Elongation at break	ASTM D638	8.3%
Flexural Strength	*ASTM D638*	65 MPa
Heat distortion temp	ASTM D638@ 0.45 MPa	88 degrees Celsius
USP Class VI Certified		No

A bite bar was manually machined on a mini-mill (Model 2000, Sherline, Vista, CA from 3/16″ Acetel rod (McMaster-Carr, Elmhurst, IL) and the ear bars were machined using 3/32″ PEEK dowel pins.

**Fig. 4:**
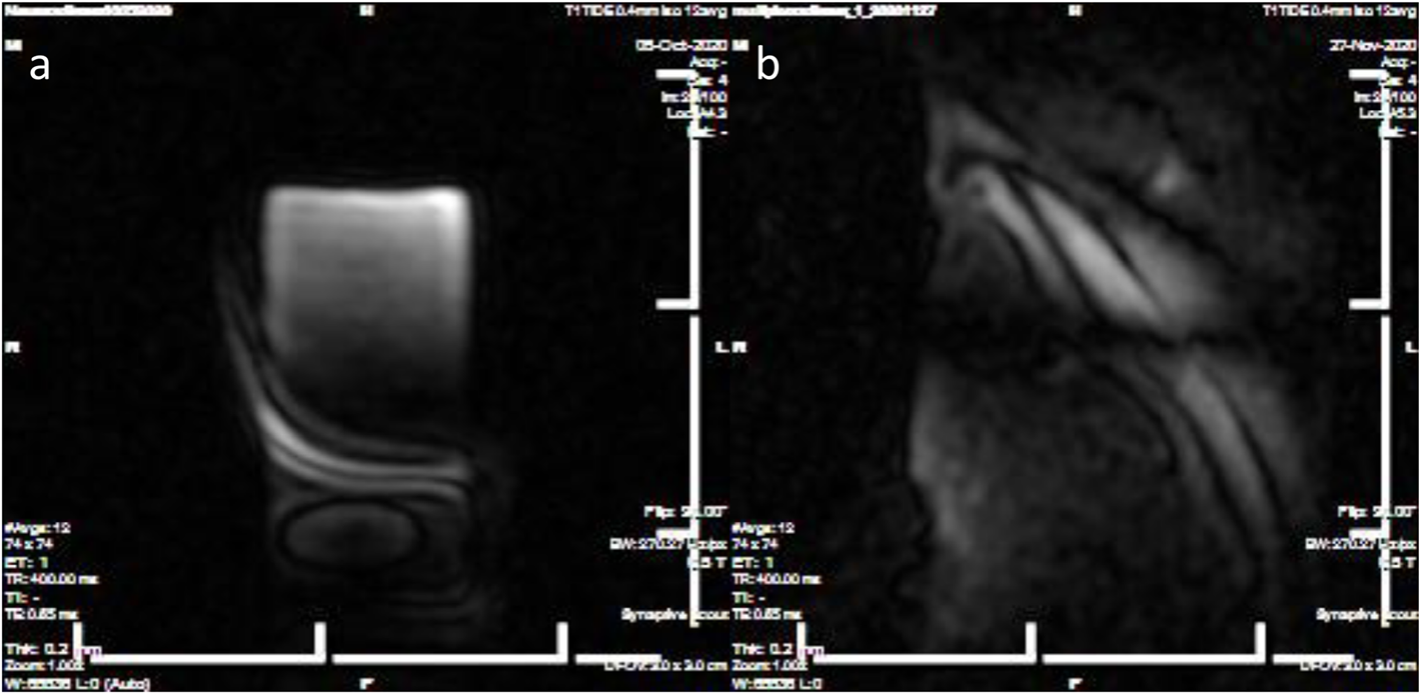
Phantom testing. **a**. Liquid filled glass ampule **b**. Polyvinyl alcohol material to simulate brain composition. Slice thickness of 0.2 mm with 12 averages; TE = 0.65 ms/TR = 400 ms

The MRI cradle was then tested in the 3-cm bore MRI system for all aspects of fit and function. To assess cradle stability, image quality and coil placement, phantom testing was performed using liquid filled vials and a viscous polyvinyl alcohol (PVA) material mixed with water that simulates brain tissue (Fig. 4). Initial imaging using routine parameters was problematic. Testing included scripts with varying slice averages to obtain resolution in the scans and assess possible artifact generation from physiologic monitoring equipment. Physiologic monitoring was done with rectal thermistor and pulse oximeter. Additional testing was done to include mouse position and fixation and anesthetic gas delivery.

### Animal models and experiments

Animal experiments were approved through the Advocate Aurora Health Institutional Animal Care and Use Committee (IACUC) following guidelines in the Guide for the Care and Use of Laboratory Animals (ILAR, NRC Academies Press). Nu/Nu female mice (Charles River, Wilmington, MA) were acclimated in the vivarium for approximately 1 week prior to testing. Mice were housed in disposable, individually ventilated caging on irradiated aspen chip bedding with a 12 h/12 h light/dark cycle. Food and water were provided ad libitum. Room temperature and humidity were maintained and monitored daily.

Surgical procedure: Mice were injected with A375 malignant melanoma cell line (ATCC, Manassas, VA) with a luciferase label. Each mouse was inoculated intracranially with cells in sterile media using orthotopic technique as described below. Mice were anesthetized with 2.2–2.4% isoflurane anesthesia in room air and maintained at 2.2% during procedures. (Somnosuite, Kent Scientific Corporation, Torrington, CT). Temperature, oxygen saturation, and heart rate were monitored intraoperatively with rectal thermistor and mouse pulse oximeter. Cells were delivered intracranially with the use of a stereotaxic platform affixed with drill and microinjector. A 1-mm burr hole was drilled approximately 1 mm anterior and 1.5 mm lateral to the right relative to bregma. Melanoma cells (1 × 10^4^ cells in 5 μl volume) were delivered at a depth of 3.5 mm with the use of a microinjector fitted with a 10-ul Hamilton syringe. Cells were delivered over a 5-min time frame, the syringe stayed in position for another 5 min, then was manually withdrawn over 5 min to prevent cell diffusion. Two additional animals underwent right flank subcuticular injections of 1 × 10^6^ cells in Matrigel Extracellular Matrix (Corning Life Sciences, Inc. Corning, NY) under sterile conditions. Post-operative monitoring included observation for neurological symptoms such as paralysis or circling. An NSAID analgesic, Carprofen (Rimadyl 5 mg/kg, Zoetis, Parsippany NJ), was administered subcutaneously immediately post-procedure and at 24- and 48-h post-procedure. Mice were weighed, and flank tumor measurements were taken twice per week.

### Image acquisition

#### MRI

**Fig. 5:**
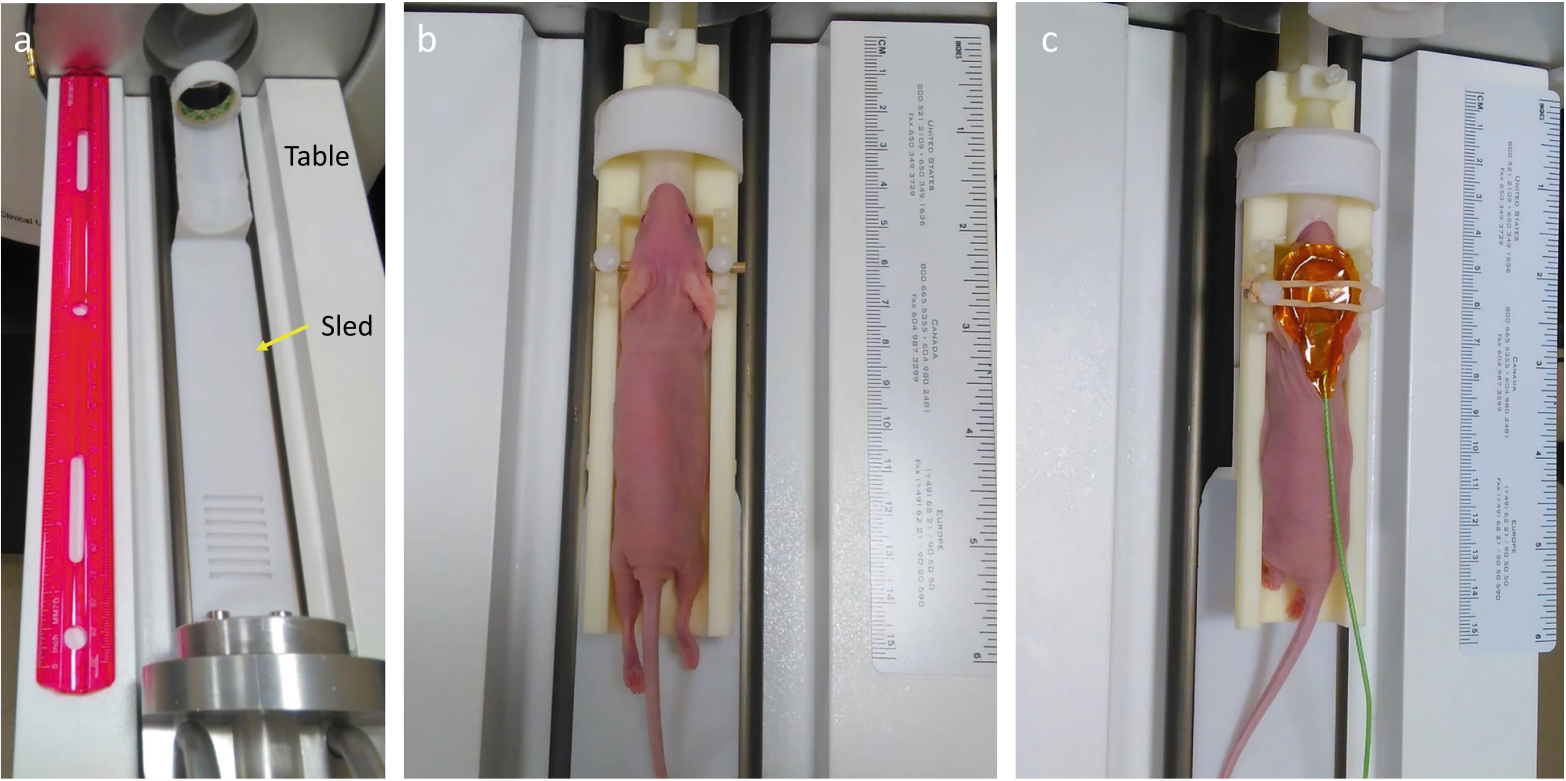
0.5 T Scout sled and table. **a**. Table and movable sled approximately 30.5 cm in length. Sled is 3 cm in width. **b**. Anesthetized mouse in cradle fitting within the 3 cm sled. Inner cradle width is 2 cm with 0.5 cm walls . **c**. Anesthetized mouse in cradle anesthetized with single channel head coil in place

Prior to use in animal imaging, the cradle was thoroughly cleaned using HB Quat detergent to wash away resin particulates which could remain after printing. Under Isoflurane anesthesia, as in the surgical procedures, mice were imaged with a 0.5 T Scout MRI beginning on day eight post-inoculation, then weekly for 4 weeks. Gadobutrol (0.1 mmol/ml, Bayer Healthcare Pharmaceuticals Inc., Whippany NJ) was injected intraperitoneally approximately 20 min prior to image acquisition to generate an enhanced T1 image with a single channel head coil (image parameters: TE = 0.65 ms /TR = 400 ms Fig. 5). Temperature was monitored with rectal thermistor and warming was provided by warm air circulation (Bair Hugger, 3 M, St. Paul MN). Oxygen saturation and heart rate were monitored through use of a pulse oximeter. As in the liquid ampule experiment, mice were scanned with varying slice averages.

#### In vivo imaging

**Fig. 6:**
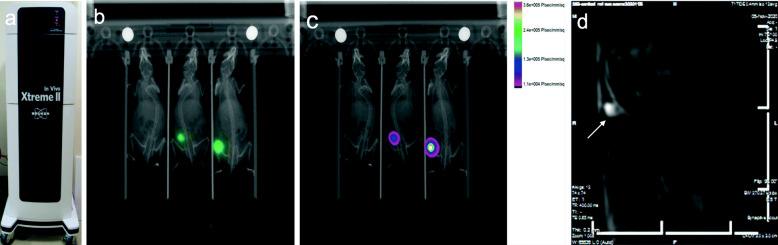
Bruker Xtreme II In vivo images. **a**. Bruker Extreme II imager **b**. Mouse luminescent images of sub-cutaneous tumors 9 days post cell injection. **c**. Color overlay showing tumor cell intensity. Images captured 20 min after administration of D-luciferin. **d**. 0.5 T Scout MRI image of sub-cutaneous tumor (white arrow) of mouse with 2 channel body coil in 3D printed cradle

In vivo images were acquired from mice injected with luciferase-tagged cells beginning at day nine post inoculation with the Xtreme II In Vivo imager (Bruker, Billerica, MA). D-Luciferin (Gold Biotechnology, St. Louis, MO) was prepared according to manufacturer recommendations and injected intraperitoneal 15 min prior to anesthesia induction. Images were acquired approximately 20 min post D-Luciferin administration with animals positioned on their backs (Fig. 6).

## Results

Our cradle design incorporates inhalation gas anesthesia and allows for the use of external monitoring devices while decreasing the amount of time for placing the animal within the imager. This represents an overall reduction in procedure anesthesia time in addition to imaging time. The M3-X material was non-reactive when scanned alone using the same parameters as the liquid filled phantom. Liquid filled ampules were scanned at 0.2 mm slice thickness with 12 and 16 averages. Differences were noted in signal to noise (SNR) when imaging the liquid filled phantom, with and without monitoring equipment. SNR is the measure of signal intensity divided by the background of an image (S/N) to achieve a ratio. Visual assessment of the images showed more noise within the images taken with 16 slices. The mock brain phantom imaged in the absence of monitoring demonstrated an SNR similar to mice scanned with monitoring. This may be due, in part, to the composition of the material as well as the size of the phantom itself in a 50-mlcentrifuge tube (Table 2).

**Table 2:** Image testing of 3D cradle printed with M3X material. Phantom and mouse testing done with and without physiologic monitoring equipment with single channel head coil

Liquid Phantom	12 slice average	16 slice average
No circulated heat or monitoring	*6.8*	1.68
Heat only	2.53	1.83
Circulated heat and monitoring	4.6	4.8
**Brain phantom**		
PVA material	7.6	
**Mice**		
Mouse a	7.8	7.97
Mouse b	6.2	6.32

**Fig. 7:**
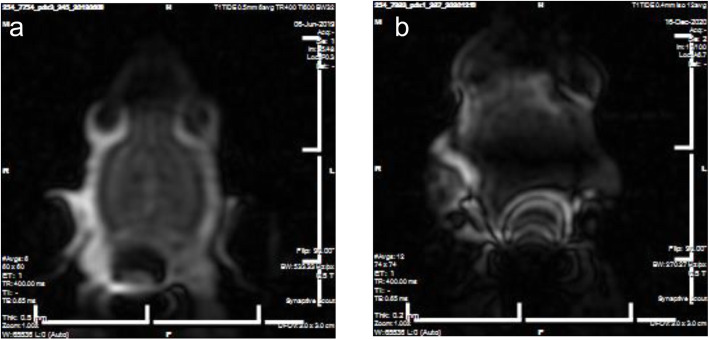
Comparison MRI images. **a**. Axial MRI image of mouse head using 50 ml centrifuge tube as a cradle and tape showing low resolution, blurry edges. **b**. Axial MRI image of mouse head using 3D-printed cradle showing improved resolution, defined edges

Imaging using the 3D printed cradle provided higher quality results compared to the handmade device previously employed (Fig. 7).

**Fig. 8:**
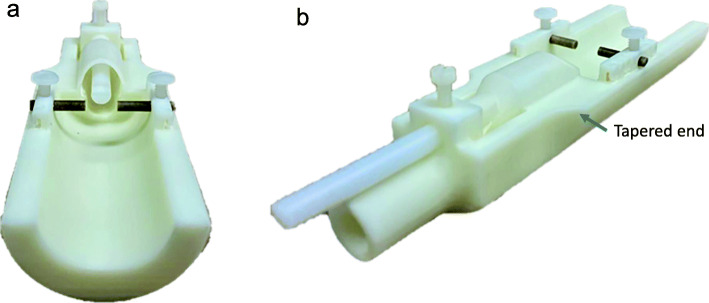
Dual view of 3D printed cradle. **a**. Ear bars, bite bar, and anesthesia nose cone with set screws. **b**. Side view with set screws, bite bar, anesthesia tunnel and tapered end allowing a seamless fit into existing sled

Since the modular design of the cradle accommodated multiple coil configurations (Fig. 8), several images could be rapidly repeated without moving the mouse during coil change.

In addition, serial images were obtained that enabled reliable assessment of tumor growth over time. Overall procedure time (set up and scan time) was reduced by 15 min; from 1 h to 45 min. This, in turn, reduced the time animals were under anesthesia. Using the 3D printed cradle, the mouse was better secured than in the original cradle apparatus, leading to improved image resolution with less movement artifact. Additionally, in validation imaging with the liquid-filled glass ampule and mice, the material itself was non-reactive and exhibited no artifact. There was no measurable warping of the printed part and the final dimensions of the prototype were highly accurate with a measured deviation of less than 0.12 mm in any dimension compared to the design dimensions. M3-X is not FDA approved for medical procedures, however for our research study, it performed to expectation and improved MRI images. The nominal cost of 3D printing was essential for our laboratory.

## Discussion

3D printing presents an opportunity to improve healthcare and pre-clinical science. From the use of substrates like plastics and metal to bio-ink for use in tissue and wound repair [13, 14], the advantages are numerous. It offers an opportunity to improve diagnostic imaging as part of a brain tumor research program. The 0.5 T Scout mouse specific MRI has limited space within a 3 cm bore which prevented purchase of an “off the shelf” cradle for animal imaging. This presented an opportunity to design an MRI-safe component to allow for mouse restraint and enable consistent, reliable images. Unlike other designs, our cradle features a tapered end to fit into an existing sled. Other laboratories have fabricated iterations of a circular tube that embed cardiac monitoring, surface RF coils and lines which circulate warm water, or cylindrical head holders which incorporate a bite bar [15, 16]. Using the limited space within a 3-cm bore, the tapered end was critical for the delivery of inhalation anesthesia.

Some advantages of the proprietary white M3-X, used to print the initial cradle, are durability, washability with mild detergents, and short print time (approximately 21 h using the 3D printer described here). One disadvantage noted for this cradle is that M3-X has a temperature resistance less than 88 °C, which does not allow sterilization using an autoclave. This is considered a detriment as experimental mice are immunocompromised and need an environment as sterile as possible to avoid infection. Ethylene oxide sterilization may be an alternative, however it was not tested as part of this study. Quaternary disinfectant detergent (HB Quat) or 70% ethanol are used as an alternative.

The cradle developed in this study is unique in that the dimensions required to fit the bore of the 0.5 T MR unit were not available in any commercially available cradle. The M3-X cradle performed well in the 0.5 T, however, future testing would include imaging in a higher field strength MRI. While integrated monitoring such as respiratory sensors or circulated water tubing were not included in the design used in this study, the design could tailored to incorporate such features and accommodate any circumstances that arise.

Although beyond the scope of this study, it is worth noting that more studies in the literature use high field strength MRI, in small experimental animals such as mice, compared to lower field strength [17–19]. For various reasons including economy, lower field strength could offer more flexibility in imaging small animals [20]. Field strengths as low as 0.1 T are portable enough to install on a tabletop [21] and because of its portability, low field MRI is frequently used for orthopedic and diagnostic imaging in other animals [22].

New product introduction may require modification of existing protocols, particularly with the use of MRI. The 3D printed cradle, with a thickness of 5 mm, altered the mouse position in the anterior/posterior (AP) plane of imaging. Therefore, AP geometry modifications were made in the acquisition program for image realignment and required collection of multiple images to determine the best AP distance. For example, using the 50 ml centrifuge tube, the AP measurement was 0 mm, while optimal imaging with the new cradle resulted in very different AP acquisition geometry. Despite some of the drawbacks or optimization required when upgrading devices, 3D printing offers multiple avenues for improving science while at the same time decreasing economic impact. For those who have needs that are not addressed by commercial products, 3D printing offers various solutions at low cost, delivered reliable and reproducible images and was a viable option for our laboratory.

## Data Availability

Data used in this study are available from the corresponding author on reasonable request.

## Abbreviations

CT: Computed tomography
RF: Radiofrequency
AP: Anterior/posterior
CAD: Computer Aided Design
3D: Three dimensional
HB Quat: hospital-grade disinfectant cleaner
IACUC: Institutional Animal Care and Use Committee
MRI: Magnetic Resonance Imaging
MJP: Multi-Jet Printing
PVA: polyvinyl alcohol
